# In Silico Screening and Development of Microsatellite Markers for Genetic Analysis in *Perca fluviatilis*

**DOI:** 10.3390/ani12141809

**Published:** 2022-07-15

**Authors:** Pu Xu, Cuiyun Lu, Zhipeng Sun, Youyi Kuang, Dingchen Cao, Tangbin Huo, Chao Li, Hongyu Jin, Xianhu Zheng

**Affiliations:** 1 National and Local Joint Engineering Laboratory for Freshwater Fish Breeding, Heilongjiang River Fisheries Research Institute, Chinese Academy of Fishery Sciences, Harbin 150070, China; 18838269885@163.com (P.X.); lucuiyun@hrfri.ac.cn (C.L.); sunzhipeng@hrfri.ac.cn (Z.S.); kuangyouyi@hrfri.ac.cn (Y.K.); caodingchen@hrfri.ac.cn (D.C.); huotangbin@hrfri.ac.cn (T.H.); lichao@hrfri.ac.cn (C.L.); jinhongyu@hrfri.ac.cn (H.J.); 2 Key Laboratory of Freshwater Aquatic Germplasm Resources, Ministry of Agriculture and Rural Areas, College of Fish and Life Science, Shanghai Ocean University, Shanghai 201306, China

**Keywords:** *Perca fluviatilis*, microsatellite markers, genetic diversity

## Abstract

**Simple Summary:**

*Perca fluviatilis* is an economically important species of freshwater fish that has flavorsome meat with a high nutritional value. Microsatellite markers are widely used in the genetic structure analysis of aquatic animals due to their abundance, high polymorphism, and codominance. In this study, we screened, tested, and developed polymorphic markers and evaluated the genetic diversity of the main wild *P. fluviatilis* populations in China. From the *P. fluviatilis* genomic data, 98,425 pairs of microsatellites were identified. A total of 200 primer pairs for tetranucleotide microsatellites were synthesized and tested in randomly selected wild individuals. Among them, 152 microsatellite markers were found to be polymorphic. A total of 29 markers with clear amplified bands and high polymorphism were selected for the genetic analysis of the four populations. The results indicated a high level of genetic diversity in *P. fluviatilis* populations in Wulungu Lake, Jili Lake, and the Wulungu River and a medium level of genetic diversity in the Kalaeerqisi River. There was moderate genetic differentiation among the populations in Xinjiang. The results of this study provide important information on the genetic diversity and genetic population structuring of *P. fluviatilis* in China as well as a scientific basis for the protection and molecular marker-assisted breeding of *P. fluviatilis*.

**Abstract:**

*Perca fluviatilis* is an economically important species of freshwater fish. To understand the genetic structure of *P. fluviatilis* in China, 268 samples were collected from Wulungu Lake (WL), Jili Lake (JL), the Wulungu River (WR), and the Kalaeerqisi River (KR). These samples were then analyzed using microsatellite markers. A total of 98,425 microsatellite markers were developed based on the genomic data, and 29 polymorphic microsatellite markers were selected to analyze genetic diversity in this study. The number of alleles (*N_a_*) and observed heterozygosity (*H_o_*) per population ranged from 4.621 (KR) to 11.172 (WL) and from 0.510 (KR) to 0.716 (JL), respectively. The results of the polymorphic information content (*PIC*) showed that the WL, JL, and WR populations were highly polymorphic (*PIC* *≥* 0.5) and that the KR population was moderately polymorphic (0.25 ≤ *PIC* < 0.5). The genetic differentiation coefficient (*F_st_*) among the four *P. fluviatilis* populations was 0.074, indicating moderate genetic differentiation among the populations in Xinjiang. The reason for the significant difference between the rivers and lakes could be the presence of a dam blocking the flow of *P. fluviatilis*. The development of microsatellite markers provides support for population genetics in the future. The evaluation of the genetic structure of *P. fluviatilis* in Xinjiang provides a reference for the reproduction and conservation of *P. fluviatilis*.

## 1. Introduction

*Perca fluviatilis* belongs to the Percidae family, part of the Perciformes order. It is commonly known as Eurasian perch and is widely distributed in Europe and North Asia [1]. It is an important species of freshwater fish, favored by farmers because of fast growth and strong disease resistance [2], and it is favored by consumers due to its flavorsome meat and high nutritional value. To date, research on *P. fluviatilis* has focused on biological characteristics [3,4], artificial reproduction [5,6], embryonic development [7], parasites [8,9], and feeding [10,11].

Some studies concerning the genetic diversity of the species have been also conducted [12,13,14]. As higher diversity implies greater adaptability to environmental changes [15], it may be a prerequisite for the survival and development of organisms. The genetic diversity of *P. fluviatilis* has previously been demonstrated to be low, based on mitochondrial control region sequencing of wild populations in Xinjiang and other breeding populations in China [16,17]. Nesbø et al. [14] also used mitochondrial control region sequences and random amplified polymorphic DNA (RAPD) markers to evaluate genetic variation in Europe and Siberian *P. fluviatilis* populations and demonstrated a high level of genetic diversity. Microsatellite markers, also known as simple sequence repeats (SSRs), are widely used in the genetic structure analysis of aquatic animals due to their abundance, high polymorphism, and codominance [18,19]. Khadher et al. [20] used twelve microsatellite markers to analyze *P. fluviatilis* in seven locations in Lake Geneva; the results showed that the number of alleles (*N_a_*) ranged from 3.33 to 4.75, reflecting low genetic diversity. Recently, Sipos et al. [21] developed twelve new polymorphic microsatellites and found that the *P. fluviatilis* genetic diversity of two Hungarian populations (*N_a_* = 8.667 and 9.500) was lower than that of a Polish population (*N_a_* = 10.667). Information regarding genetic diversity is limited; it is, therefore, necessary to use a greater number of markers to evaluate the current genetic diversity of *P. fluviatilis.* It is of significance to evaluate the genetic resources for the reproduction and protection of *P. fluviatilis*.

Nowadays, the development of sequencing technology has led to a rapid increase in high-throughput sequencing data in public databases [22]. Genome sequencing data are frequently used for the development of SSR markers for a wide range of species [23,24,25], and several whole-genome assemblies of *P. fluviatilis* have been published recently [26]. Therefore, we used the *P. fluviatilis* genome data for the screening, characterization, and testing of microsatellite markers. In China, *P. fluviatilis* is mainly distributed in the Altay Region, Xinjiang [27]. After successful artificial propagation, many provinces have introduced wild parents from this region for cultivation. However, these wild *P. fluviatilis* populations lack data regarding their genetic diversity. We also used the new markers to evaluate the genetic diversity of four wild populations in Xinjiang, providing a scientific basis for the reasonable protection and molecular marker-assisted breeding of *P. fluviatilis*.

## 2. Materials and Methods

### 2.1. Sample Collection

**Figure 1:**
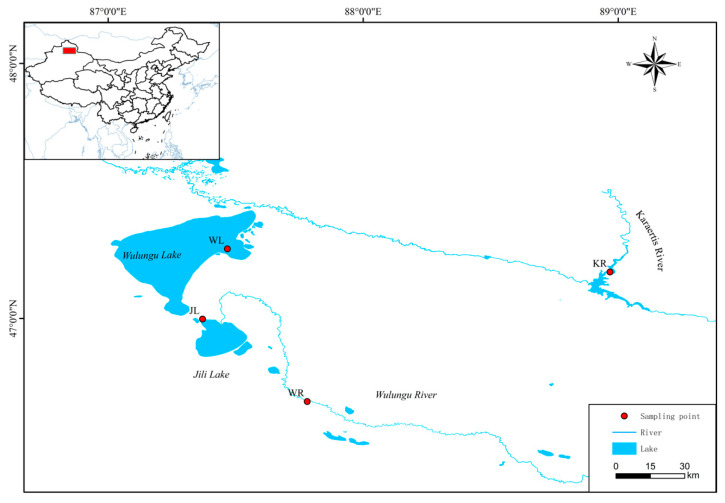
Sampling distribution of four *P. fluviatilis* populations. WL: Wulungu Lake; JL: Jili Lake; WR: Wulungu River; KR: Kalaeerqisi River.

A total of 268 samples were collected from the Altay region of Xinjiang, China, including 34 samples from Kalaeerqisi River (KR), 77 samples from Wulungu River (WR), 68 samples from Jili Lake (JL), and 89 samples from Wulungu Lake (WL) (Figure 1). Approximately 1 cm^2^ of the caudal fin of each sample was collected and pasted onto filter paper and another absorbent paper, covered with another filter paper, and kept at room temperature after drying naturally [28] for later use. The fish were released back into the capture sites after sampling.

### 2.2. Primer Design and Screening

The genome data of *P. fluviatilis* were downloaded from the GenBank database (accession number: GCA_010015445.1), and the microsatellites were isolated and screened using MISA software (https://webblast.ipk-gatersleben.de/misa/) (accessed on 1 May 2021). According to standards such as those reported by Becker and Heun [29], the search criteria used to detect the primers were: more than five dinucleotide repeats, more than four trinucleotide repeats, more than three tetranucleotide repeats, and more than two pentanucleotide repeats. Primer software (version 3.0) [30] was used to design the microsatellite primers based on product size in the range of 50–500, primer size in the range of 18–30, and melting temperature in the range of 52–65 °C. A total of 200 pairs of microsatellite primers were synthesized by Jinweizhi Biotechnology Co., Ltd., Suzhou, China.

### 2.3. DNA Extraction and PCR Amplification

Genomic DNA was extracted from the caudal fin using a standard proteinase K-phenol-chloroform extraction protocol [31]. A NanoDrop^TM^ 8000 spectrophotometer (Thermo Fisher Scientific, lnc., Wilmington, DE, USA) was used to detect the DNA concentration and optical density value; 1.5% agarose gel electrophoresis was used to detect the integrity. Finally, the samples were diluted to 50 ng/μL and stored in the refrigerator at −20 °C for later use.

Four samples from different populations were randomly selected to preliminarily detect the polymorphism and specificity of the 200 pairs of primers [32]. Finally, 29 pairs of polymorphic primers were selected to analyze all samples (Table 1). The DNA samples were amplified by PCR in a total reaction volume of 15 μL, which contained 1 μL of genomic DNA (50 ng/μL), 7.5 μL of 2 × PCR MIX (Shanghai Yisheng Biotechnology Co., Ltd., Shanghai, China), 0.3 μL of forward and reverse primers (10 μM), and 5.9 μL of ddH_2_O. Each primer pair was amplified separately. The PCR parameters were as follows: initial denaturation at 95 °C for 3 min followed by 35 cycles of denaturation at 94 °C for 20 s; annealing at 58 °C for 30 s; extension at 72 °C for 40 s; and a final extension at 72 °C for 5 min. An amount of 1 μL of the product was then obtained and mixed with 9 μL of loaded HIDI, denatured at 95 °C for 3 min, immediately bathed in ice water, and placed in an ABI 3730XL (Applied Biosystems, Foster City, CA, USA) sequencer for electrophoresis detection. GeneMarker software was used for the data analysis.

### 2.4. Data Analysis

PopGene version 3.2 [33] was used to calculate the number of alleles (*N_a_*) and the number of effective alleles (*N_e_*) per locus as well as the observed heterozygosity (*H_o_*) and expected heterozygosity (*H_e_*). Using the formula of Bostein et al. [34], we calculated the polymorphic information content (*PIC*) per locus. We also used PopGene version 3.2 to evaluate the Hardy–Weinberg equilibrium test (*P*_HWE_) and genetic distance (*D*) between the populations [33]. To construct an unweighted pair-group method with an arithmetic mean (UPGMA) dendrogram, PHYLIP version 3.6 software (https://evolution.genetics.washington.edu/phylip.html) (accessed on 12 October 2021) was used. Arlequin version 3.5 software [35] was used to evaluate the source of variation (AMOVA) and genetic differentiation coefficient (*F_st_*) based on the premise that the number of permutations was 1000. The genetic composition within the populations and differences among them were analyzed using Structure software version 2.3.4 [36]; the range of clusters (K) was predefined from 1 to 7 with 15 independent runs for each K. For each run, an MCMC chain length of 50,000 burn-in iterations and 100,000 sampling iterations was used. The most probable K value was selected using Structure Harvester (http://taylor0.biology.ucla.edu/structureHarvester/) (accessed on 15 October 2021).

## 3. Results

### 3.1. Analysis of the Sequence Characteristics of Microsatellites in the P. fluviatilis Genome

**Figure 2:**
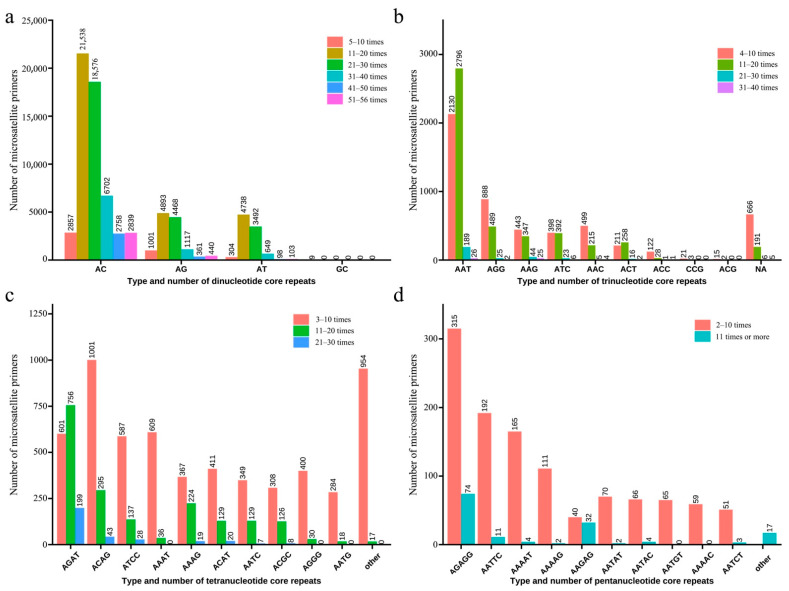
Distribution of microsatellite repeat motif types in the *P. fluviatilis* genome. Different colors represent the repeat times of different core sequences. (**a**) Type and number of dinucleotide core repeats. (**b**) Type and number of trinucleotide core repeats. (**c**) Type and number of tetranucleotide core repeats. (**d**) Type and number of pentanucleotide core repeats.

From the *P. fluviatilis* genomic data, 98,425 microsatellite markers were developed. The detailed primer information is presented in Supplementary Table S1. Dinucleotide repetitive microsatellites (76,943; 78.17%) appeared most frequently (Figure 2), including the (AC)_n_, (AG)_n_, (AT)_n_, and (GC)_n_ repeat motif. The (AC)_n_ core repeat type had the highest number and the (CG)_n_ had the lowest number. The trinucleotide-repeat microsatellite loci (10,494; 10.66%) included (ATT)_n_, (AGG)_n_, (AGC)_n_, (AAG)_n_, (ATC)_n_, (AAC)_n_, and (ACT)_n_, with a total of 10 types of repeat motif; among them, the number of (ATT)_n_ was the highest. The tetranucleotide-repeat microsatellite sequence included 32 repeat motifs (Table 2), of which the (AGAT)_n_ and (ACAG)_n_ repeat motif appeared most frequently, accounting for approximately 17.78% and 15.3%, respectively. The pentanucleotide repeat (2237; 2.27%) was the least frequent, including 94 core repeat motifs of (AGAGG)_n_, (AATTC)_n_, (AAAAT)_n_, and (AAAAG)_n_.

### 3.2. Polymorphisms of the Microsatellite Markers

A total of 200 primer pairs for tetranucleotide microsatellites with a repeat motif of (AGAT)n or (ACAG)n were tested in four wild individuals. The information on all 200 tested primers is presented in Supplementary Table S2. The results showed that 191 microsatellite markers had clear bands, accounting for 95.5%. Among them, 152 microsatellite markers were found to be polymorphic, accounting for 76%.

A total of 29 markers with clear amplified bands and high polymorphism were selected for the genetic analysis of the four populations. A total of 364 alleles were amplified in 268 samples from four *P. fluviatilis* populations, with fragment sizes ranging from 86 to 265 bp. The *N_a_* per locus ranged from 4 (HLJHL052 and HLJHL007) to 29 (HLJHL186), with an average of 12.552. The *N*_e_ per locus ranged from 1.313 (HLJHL084) to 10.913 (HLJHL186), with an average of 4.809. The *H*_o_ per locus ranged from 0.896 to 0.234 (an average of 0.648) and the *H*_e_ ranged from 0.910 to 0.237 (an average of 0.712). The *PIC* ranged from 0.225 to 0.901, with an average of 0.680. Of these markers, 23 were highly polymorphic markers (*PIC* ≥ 0.5); HLJHL186 had the highest *PIC* (Table 3).

### 3.3. Genetic Diversity of P. fluviatilis Populations

The results on the genetic diversity of the four *P. fluviatilis* populations are shown in Supplementary Table S3. The WL population had the highest *N_a_* (11.172) and *N_e_* (5.018). The KR population had the lowest *N_a_* (4.621) and *N_e_* (2.563). The JL population had the largest *H_o_* (0.716) and *H_e_* (0.749), and the KR population had the lowest *H_o_* (0.510) and *H_e_* (0.518). The *PIC* ranged from 0.467 to 0.716. Based on the *PIC* results, the levels of genetic diversity among the populations were ranked: JL > WL > WR > KR. The JL, WL, and WR populations were highly polymorphic (*PIC* > 0.5), whereas the KR population was moderately polymorphic (0.25 < *PIC* < 0.5). The WR, JL, WL, and KR populations had 8, 7, 8, and 4 loci, respectively, showing a departure from the Hardy–Weinberg equilibrium, with a significant or an extremely significant lack of heterozygotes.

### 3.4. Genetic Differentiation of P. fluviatilis Populations

**Figure 3:**
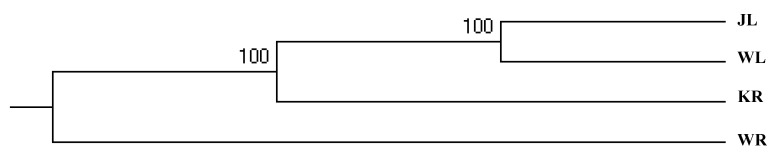
UPGMA dendrogram of four *P. fluviatilis* populations based on the unbiased genetic distance of *N_e_*.

The AMOVA results showed that 7.44% of the genetic variation was among the populations (Table 4). The *F_st_* value for the four *P. fluviatilis* populations was 0.074, representing a moderate level (0.05 < *F_st_* < 0.15). The pairwise *F_st_* was significant for the lake vs. the river samples in the JL–WL–WR basin (0.05 < *F_st_* < 0.15), representing a moderate level (Table 5). The *F_st_* between the rivers (KR and WR) (*F_st_* = 0.158) was the highest, representing high-level genetic differentiation (*F_st_* > 0.15). The *F_st_* (*F_st_* < 0.05) was lower between the two lakes, despite the significant differences. Correspondingly, the genetic distance between the two rivers was the largest (*D* = 0.288). The genetic distance between the two lakes (JL and WL) was the closest (*D* = 0.027) (Table 5). Based on the cluster tree (Figure 3), it was clear that the four populations of *P. fluviatilis* were clustered into three groups: the JL and WL populations belonged to one branch, the KR population belonged to another branch, and the WR population belonged to a third branch.

### 3.5. Analysis of the Population Genetic Components

**Figure 4:**
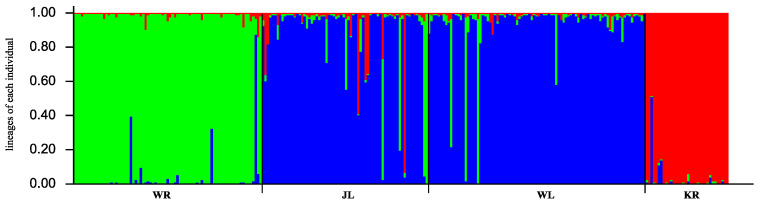
Estimated population genetic structure in which each vertical line represents an individual, different colors represent different genetic components, and the populations are separated by black lines (K = 3). The populations are labeled below the figure.

According to the results from Structure Harvester, K = 3 was the best value to cluster the four populations because the delta K value was the largest. The four populations could then be classified into three genetic components (Figure 4): the WR population constituted one group, the KR population constituted another group, and the WL and JL populations were clustered into one group.

## 4. Discussion

### 4.1. Characteristics and Screening of P. fluviatilis Microsatellite Sequences

Among the 98,425 microsatellite loci in the *P. fluviatilis* genome, the dinucleotide repeats were the most abundant, which is consistent with most vertebrates [37,38], such as *Sander lucioperca* [39], *Megalobrama amblycephala* [40], and *Lateolabrax maculatus* [41]. Among the dinucleotide repeat sequences, the (AC)_n_ core repetitive types accounted for the highest proportion, which is similar to the findings for *Tetraodontidae* [42]. The low content of the (CG)_n_ core repeats is also consistent with that of most species. Schorderet and Gartler [43] believed that this phenomenon was caused by the methylation and deamination of cytidine C into thymine T through CpG.

The tetranucleotide-repeat microsatellite markers showed a higher level of polymorphism and greater stability than the dinucleotide repeats and trinucleotide repeats and are more suitable for analyzing the genetic differences between the populations [44]. In this study, among the tetranucleotide-repeat microsatellite loci, (AGAT)_n_ was the most abundant (17.78%). This may have been due to the specificity of the species, as it is contrary to the research findings of Zheng et al. [45]. There were also differences in the repeat types of the tetranucleotide microsatellites among the different species. For example, in the *Pinctada martensii* EST database, (ATTT)_n_ accounted for 50% of the tetranucleotide-repeat microsatellites [46]. Among the 29 pairs of microsatellite primers of *P. fluviatilis* used in this study, the core sequence was repeated more than 10 times and the number of alleles at each locus was not less than 4. Barker [47] pointed out that when microsatellite markers are used for genetic analyses, the number of alleles should not be less than four and those with fewer than four bands should be excluded. Therefore, the primers in this study could provide correct genetic information for the study of the population genetics of *P. fluviatilis.*

### 4.2. Genetic Diversity and Differentiation of P. fluviatilis Populations

Genetic diversity is not only a prerequisite for biological survival and development but also an important basis for assessing the status of genetic resources [48]. It can be determined based on indicators, such as *N_a_*, *H_o_*, and *PIC* [49]. In this study, the genetic diversity of the two lakes was higher than that of the two rivers. In its early years, *P. fluviatilis* in the lake probably came from the Eerqisi River basin, which resulted in a greater genetic source in the lakes. Therefore, the genetic diversity of *P. fluviatilis* in the lakes was higher than that in the rivers of the WL–JL–WR basin. The water in the lake was more stable and, therefore, more suitable for spawning [50]. The KR population was the lowest based on the *N_a_*, *H_o_*, and *PIC* parameters. The Kalaeerqisi River is an upstream tributary of the Eerqisi River; *P. fluviatilis* migrated upstream from Europe to China via the Eerqisi River, so the *P. fluviatilis* genetic resources of the KR population could not be better supplemented. Compared with the results of Yang et al. [51], the *H*_o_ and *H*_e_ of the Wulungu Lake were lower in this study, showing that the genetic richness of *P. fluviatilis* decreased and that the germplasm resource has deteriorated over the past years. Compared with *P. fluviatilis* around Lake Geneva, the *N_a_*, *H_o_*, and *H_e_* of the four populations in this study were higher [20], indicating that the genetic diversity of wild *P. fluviatilis* in China was higher than in Lake Geneva.

The genetic differentiation index (*F_st_*) is an important parameter to describe population differentiation. The larger the *F_st_* value, the greater the genetic distance and the more distant the genetic relationship between the populations. Wright [52] believed that *F_st_* < 0.05 indicated low genetic differentiation, that 0.05 < *F_st_* < 0.15 indicated moderate genetic differentiation, and that 0.15 < *F_st_* < 0.25 indicated high genetic differentiation. The *F_st_* among the four populations in the four regions of the Wulungu River, Wulungu Lake, Jili Lake, and the Kalaeerqisi River was 0.074, showing moderate genetic differentiation, probably due to the geographical proximity of the four regions. There was significant genetic differentiation among the different river basins. It is noteworthy that the highest degree of genetic differentiation was observed between the KR and WR populations. In 1969, the “Diversion Eji Lake” project introduced the Eerqisi River into the Wulungu Lake to reduce the salinity of the internal lake [53]; at the same time, fish entered the lake along the river [54]. This could have weakened the genetic differentiation. Therefore, the genetic differentiation between the KR and WL or JL populations was less than that between the KR and WR populations. The genetic differentiation among the river and lake populations in the Wulungu River basin reached a moderate level, with a significant river–lake differentiation. Although the water of the Wulungu River flows into Wulungu Lake through Jili Lake, a dam blocks the movement of *P. fluviatilis* from the river into the lake, causing geographical isolation, leading to the existing genetic differentiation between the rivers and lakes within the basin. There is no geographic barrier between Wulungu Lake and Jili Lake, which are connected by the Kuiga River. It was also seen from the AMOVA results that only 7.44% of the genetic variations occurred between the populations, while approximately 92.56% occurred within the populations. The UPGMA cluster tree was divided into three branches; this result was consistent with the structured genetic components.

## 5. Conclusions

*Perca fluviatilis* is an economically important species of freshwater fish. The developed microsatellites can be used for genetic structure analysis; dentification of related, inbred individuals in aquaculture settings; and detection of family structure. Based on the genome data for *P. fluviatilis*, we identified and designed 98,425 pairs of microsatellite primers. A total of 200 primer pairs for tetranucleotide microsatellites were synthesized and tested in wild individuals. Among them, 152 microsatellite markers were found to be polymorphic. We selected 29 markers with clear amplified bands and high polymorphism for the genetic analysis of the four populations. The results showed that the genetic diversity of the two lakes was higher than that of the two rivers. We conclude that it is essential to protect the germplasm resources of *P. fluviatilis* in the Kalaeerqisi River so that the genetic diversity will not be reduced or lost. Over the years, *P. fluviatilis* from Xinjiang has been introduced into many other regions for artificial cultivation. Our study used microsatellite markers to distinguish the genetic differences among the different populations and has laid a foundation for cross-regional introduction.

## Figures and Tables

**Table 1:** Microsatellite primer sequences and amplification information for *P. fluviatilis*.

Locus	Forward Primer Sequence (5′–3′)	Reverse Primer Sequence (5′–3′)	Repeat Motif	Fragment Size/bp
HLJHL007	GCTAATGCCTGACCACCACT	CAGGTCCCTGGAGAAGAGAGA	(ACAG)_10_	109–130
HLJHL022	CCCTGAGTGGAGCACATACC	GGCCGAATTTTGCCTTTGTA	(ACAG)_12_	100–131
HLJHL045	GATGGTCTCACGAGGCTAGC	TCGGTTTGGATGGTCGGTTT	(ACAG)_14_	86–114
HLJHL052	ACCTGAAGGCAAGTGGATGG	CCTGGCCCTGAATGTACCAA	(ACAG)_15_	92–120
HLJHL056	CAGCAGCCTGATCAGCATCA	TGCTTTACTTCCTCATCACAGC	(ACAG)_15_	120–148
HLJHL059	GGTTTCAAGGGAGAGGGAGG	GCAGCTCATGAACAACTCTGC	(ACAG)_15_	92–145
HLJHL084	AGCTCGACTAGGGTGACTGT	CCGAGCACTGCTACGAAACT	(ACAG)_18_	143–174
HLJHL089	ATTCACACAAACAAGCAGGC	GCTATGTGGCTCTTTGTATGCC	(ACAG)_19_	100–160
HLJHL090	GGCGCTGTCCATGGTACTAA	ACCACGAAAAGCAAGAAGGC	(ACAG)_19_	117–162
HLJHL094	ACTCACCATACGCATGTGCT	CTCCTCAAAGTCGCCTTCCA	(ACAG)_19_	114–186
HLJHL104	CCCAAATTTCCTGACAACCCA	GGACTGTCCCGTGTTTCTCA	(AGAT)_10_	132–223
HLJHL105	GCGCGATAAAATAATTGTCGGC	TCAGGCTCAGGAATTTCTTTCA	(AGAT)_10_	107–148
HLJHL107	AGACAGGGTGATAGTTACATCCA	TGTGCAAAATTTACATGGGATGA	(AGAT)_10_	118–188
HLJHL121	ATTGGCATCAGAGCAAGCTG	ATGGGGCTTTGACGTGAGAG	(AGAT)_11_	184–265
HLJHL152	GCCAACACCCTATAACTGAAGC	CGTTGTTGCCAATGGAATGC	(AGAT)_14_	140–185
HLJHL153	GGCTGATAAACATAGGCCTATGC	AGCTACTGGGATCTTGAAGGT	(AGAT)_14_	123–151
HLJHL164	CCACCTCTGCCACCTCATAC	CGAAGGGATCTCCATCTGCT	(AGAT)_15_	104–175
HLJHL165	TCCAGTTGTCACTTCAGCGT	AGGACACATTTCCTTCGGGA	(AGAT)_15_	88–194
HLJHL167	CGTTTTGGATATGTGCCATGT	TGGCACATATCTAAAACGTGGT	(AGAT)_15_	140–188
HLJHL169	GCAGGGGCAAACAGTCATCA	TCTGTGAGCTACTGGGACCT	(AGAT)_16_	100–152
HLJHL172	ACAGCCCATAACACAGCAGT	TCTGCATGAACTAAAGTGTGACA	(AGAT)_16_	126–187
HLJHL174	TCAGCTGCGGATTATTACACA	GCAGTGATATTGCAACAGGAAA	(AGAT)_16_	131–162
HLJHL179	GGTGATACATAGATAGGTAGGTCGG	TCTGGTAGTCTCAGCTCGCT	(AGAT)_16_	96–153
HLJHL183	TGTTGTCAGTGTGTTCATCCA	TGCATGGTGTTTTAAGTCAGGG	(AGAT)_17_	125–168
HLJHL186	CAACCAGCTTCAACCCGTTG	TCCACCTCTCCCTTTCCCTT	(AGAT)_17_	123–237
HLJHL189	CCTCCTGTGTTTTGTGTCTTGG	TCTCCAGTACTCACAATGGCT	(AGAT)_18_	124–175
HLJHL192	TGGTTCTACAAGCTGCCTAAA	AACCAGGCGTTGAGTTTCAA	(AGAT)_18_	102–168
HLJHL196	TCTGAGACAAAGGGACATGAAT	CAGGAATTTCCCCAGTGTGG	(AGAT)_19_	111–183
HLJHL199	TGGACTAAGACTGCCTACTGC	CCTTGAGTTCACTTGCGTGT	(AGAT)_20_	134–190

**Table 2:** Repeat motifs and the proportion of tetranucleotide-repeat microsatellite loci in the *P. fluviatilis* genome.

Repeat Motif Type	Count	Proportion
(AGAT)_n_	1556	17.78%
(ACAG)_n_	1339	15.30%
(ATCC)_n_	752	8.59%
(AAAT)_n_	645	7.37%
(AAAG)_n_	610	6.97%
(ACAT)_n_	560	6.40%
(AATC)_n_	485	5.54%
(ACGC)_n_	442	5.05%
(AGGG)_n_	430	4.91%
(AATG)_n_	302	3.45%
(AAGG)_n_	234	2.67%
(AAAC)_n_	221	2.53%
(AAGT)_n_	201	2.30%
(ACTC)_n_	173	1.98%
(ACTG)_n_	135	1.54%
(AACT)_n_	127	1.45%
(AGGC)_n_	107	1.22%
(AGCT)_n_	101	1.15%
(AGCC)_n_	75	0.86%
(AATT)_n_	69	0.79%
(AACC)_n_	52	0.59%
(ACCT)_n_	40	0.46%
(ACCC)_n_	37	0.42%
(ATGC)_n_	22	0.25%
(ACCG)_n_	9	0.10%
(AAGC)_n_	8	0.09%
(AGCG)_n_	7	0.08%
(ATCG)_n_	4	0.05%
(ACGG)_n_	3	0.03%
(CCCG)_n_	2	0.02%
(ACGT)_n_	2	0.02%
(CCGG)_n_	1	0.01%

**Table 3:** Parameters of polymorphism described for the 29 microsatellite loci selected in the populations of *P. fluviatilis*.

Locus	*N_a_*	*N_e_*	*H_o_*	*H_e_*	*PIC*
HLJHL007	4	2.201	0.403	0.547	0.489
HLJHL022	6	2.169	0.496	0.540	0.450
HLJHL045	8	2.133	0.485	0.532	0.496
HLJHL052	4	1.784	0.425	0.440	0.364
HLJHL056	5	2.827	0.623	0.647	0.578
HLJHL059	10	4.444	0.675	0.776	0.741
HLJHL084	9	1.310	0.239	0.237	0.225
HLJHL089	11	2.264	0.489	0.559	0.528
HLJHL090	11	1.671	0.373	0.402	0.389
HLJHL094	18	3.057	0.623	0.674	0.661
HLJHL104	17	7.506	0.869	0.868	0.853
HLJHL105	11	7.389	0.847	0.866	0.850
HLJHL107	18	9.968	0.896	0.901	0.892
HLJHL121	19	10.077	0.877	0.902	0.893
HLJHL152	13	5.949	0.791	0.833	0.811
HLJHL153	8	4.181	0.716	0.762	0.725
HLJHL164	14	5.863	0.776	0.831	0.809
HLJHL165	20	3.269	0.608	0.695	0.650
HLJHL167	13	6.486	0.519	0.847	0.827
HLJHL169	9	3.256	0.690	0.694	0.641
HLJHL172	15	4.677	0.750	0.788	0.765
HLJHL174	9	4.714	0.731	0.789	0.760
HLJHL179	11	3.273	0.612	0.696	0.658
HLJHL183	12	3.973	0.698	0.750	0.720
HLJHL186	29	10.914	0.694	0.910	0.901
HLJHL192	14	5.783	0.694	0.829	0.808
HLJHL196	15	2.389	0.526	0.583	0.512
HLJHL199	16	9.361	0.843	0.895	0.884
HLJHL200	15	6.564	0.813	0.849	0.833

Detected results for 29 microsatellite loci and the genetic structure of *P. fluviatilis* in Xinjiang. *N_a_*: allele number; *N*_e_: effective allele number; *H*_o_: observed heterozygosity; *H*_e_: expected heterozygosity; *PIC*: polymorphism index content.

**Table 4:** Results of the AMOVA based on 29 microsatellite markers.

Source of Variation	Degree of Freedom (df)	Sum of Squares	Variance Components	Percentage of Variation/%
Among populations	3	334.462	0.784	7.44
Within populations	532	5188.229	9.752	92.56
Total	535	5522.690	10.536	

**Table 5:** The genetic distance (*D*) (below-diagonal) and genetic differentiation index (*F_st_*) (above-diagonal) of four *P. fluviatilis* populations.

Population	WR	JL	WL	KR
WR	/	0.087 *	0.071 *	0.158 *
JL	0.205	/	0.003	0.116 *
WL	0.157	0.027	/	0.100 *
KR	0.288	0.244	0.1987	/

* Indicates a significant difference (*p* < 0.05).

## Data Availability

Not applicable.

## Supplementary Materials

The following supporting information can be downloaded at: https://www.mdpi.com/article/10.3390/ani12141809/s1, Table S1: A total of 98,425 pairs of microsatellite primers were identified and designed from the *P. fluviatilis* genomic data; Table S2: The result of 200 microsatellite markers evaluated by four random wild individuals in *P.fluviatilis*. Table S3: Genetic diversity parameters of 29 microsatellite markers in four Perca fluviatilis populations.
